# Comparison of clinical characteristics and antibiotic susceptibility between *Pseudomonas aeruginosa* and *P. putida* keratitis at a tertiary referral center: a retrospective study

**DOI:** 10.1186/s12886-018-0882-3

**Published:** 2018-08-20

**Authors:** Chan Ho Cho, Sang-Bumm Lee

**Affiliations:** Department of Ophthalmology, Yeungnam University College of Medicine, 170, Hyunchung-ro, Nam-gu, Daegu, 705-717 (42415) South Korea

**Keywords:** Antimicrobial susceptibility, Contact lenses, *Pseudomonas aeruginosa*, *Pseudomonas putida*, Ulcerative keratitis

## Abstract

**Background:**

To compare clinical characteristics and antibiotic susceptibilities in patients with *Pseudomonas aeruginosa* (PA) and *P. putida* (PP) keratitis at a tertiary referral center in South Korea.

**Methods:**

Forty-nine cases of inpatients with culture-proven PA and PP keratitis were reviewed retrospectively between January 1998 and December 2017. We excluded cases of polymicrobial infection. Epidemiology, predisposing factors, clinical characteristics, antibiotic susceptibilities, and treatment outcomes were compared between the PA and PP groups. The risk factors for poor clinical outcome were evaluated on the basis of the total cohort and analyzed using multivariate logistic regression.

**Results:**

A total of 33 eyes with PA keratitis and 16 eyes with PP keratitis were included. The mean age was 47.0 years in the PA group and 59.3 years in the PP group (*p* = 0.060). Differences were observed between the PA and PP groups in hypopyon (45.5% vs 6.3%, *p* = 0.006) and symptom duration (4.3 vs 9.5 days, *p* = 0.022). The most common predisposing factor for PA was wearing contact lenses (36.4%) and that for PP was corneal trauma (62.5%). No significant differences were observed in sex, previous topical steroid use, systemic disease, or duration of hospitalization between the two groups. The PA and PP groups both demonstrated good efficacy of colistin (both 100%), tobramycin (93.3%, 100%), ceftazidime (93.9%, 87.5%), and ciprofloxacin (96.6%, 87.5%). Imipenem (100% vs 81.3%, *p* = 0.030), piperacillin (96.6% vs 75%, *p* = 0.047), and ticarcillin (85% vs 0%, *p* < 0.001) showed significantly lower efficacy in the PP group than in the PA group. A poor clinical outcome was observed in 31.2% of the PA group and 37.5% of the PP group (*p* = 0.665). The risk factors for poor clinical outcome were previous ocular surface disease (odds ratio 10.79, *p* = 0.012) and hypopyon (odds ratio 9.02, *p* = 0.024).

**Conclusions:**

The PA group was more closely associated with younger age, wearing contact lenses, shorter symptom duration, and hypopyon, whereas the PP group was more closely associated with elderly age, corneal trauma, and decreased efficacy of the beta-lactams. Clinical outcomes were not significantly different between the two groups. Previous ocular surface disease and hypopyon were the risk factors for poor clinical outcome.

## Background

The *Pseudomonas* species are ubiquitous gram-negative bacteria that are major opportunistic human pathogens that cause infection, including that of the cornea. *P. aeruginosa* (PA) is one of the pathogens most destructive to the eye and is a leading cause of bacterial keratitis in contact lens (CL) wearers and those with ocular injuries [1, 2]. *Pseudomonas* keratitis progresses rapidly and is characterized by infiltration of inflammatory cells and tissue destruction. It can lead to corneal perforation and subsequent loss of vision if appropriate therapy is not promptly initiated. Therefore, culture-proven diagnosis and prompt treatment of *Pseudomonas* keratitis are crucial.

*Pseudomonas* species other than PA are considered to be less virulent than PA. This is explained by genetic differences such as the presence of DNA associated with the synthesis of protease IV [3]. *P. putida* (PP) belongs to the fluorescent group of *Pseudomonas* species, a group of opportunistic pathogens that primarily cause nosocomial infections [4]. PP is recognized as a rare cause of systemic infections, such as sepsis, mainly observed in immunocompromised patients [4, 5]. In the field of ophthalmology, there are few reports of *P. putida* keratitis [6].

Antibiotic resistance of *Pseudomonas* species has been reported to be steadily increasing over the past several decades. For example, fluoroquinolone is the drug of choice in cases of *Pseudomonas* infection; however, increasing resistance to this drug has been observed since the 1990s [7, 8]. Recently, the incidence of multidrug-resistant PA and PP isolates has been reported; hence, it is essential to evaluate periodic antibiotic susceptibility [5, 9].

The PA and PP species belong to the same genus, but there was no clinical analysis comparing between PA and PP keratitis. Therefore, we conducted a comparative study of patients with PA and PP keratitis at a tertiary referral center in South Korea. The aim of this study is to compare epidemiology, predisposing factors, clinical characteristics, antibiotic susceptibility, and treatment outcome between PA and PP keratitis.

## Methods

We conducted a retrospective, observational case series study of patients with culture-proven PA and PP keratitis at Yeungnam University Hospital in South Korea between January 1998 and December 2017. We excluded cases of polymicrobial infection and cases of outpatients. Admission decisions were based on the severity of keratitis, potential threat to vision, and need for intensive topical antimicrobial agents, and were determined by a single physician (S. B. Lee). During admission treatment, the patient was discharged if no further surgical treatment was required and complete epithelialization with sterilization were achieved.

Microbiological records and medical charts were reviewed retrospectively. We compared epidemiology, predisposing factors, clinical characteristics, antibiotic susceptibility, and treatment outcome between the PA and PP groups. Epidemiological data, including sex, age, seasonal distribution, symptom duration (defined as interval from the onset of symptoms to the time of initial presentation), and duration of hospitalization (defined as the period from admission to discharge), were reviewed. Age was divided into four subgroups (0–19, 20–39, 40–59, and ≥ 60 years). Predisposing factors, including corneal trauma, wearing CLs, previous ocular surface disease (OSD), previous ocular surgery, topical antibiotic use, topical steroid use, and underlying systemic disease, were evaluated. The categories of corneal trauma include those caused by industrial materials, foreign bodies, or vegetable matter. Clinical characteristics, including the location and size of the corneal lesion, and hypopyon were reviewed at initial presentation. Corneal lesions were divided into central and peripheral lesions according to their location on the basis on the middle point of the corneal radius. The size of the corneal lesion was based on the size of the corneal epithelial defect with estimation of the area of an equivalent rectangle. The largest linear dimension of the epithelial defect and its largest possible perpendicular within the confines of the epithelial defect were measured using the ruler of a slit lamp biomicroscope [10].

Before therapy was initiated, corneal scrapings of all cases were obtained using a No. 15 Bard-Parker knife (Aspen Surgical, Caledonia, MI, USA) after application of 0.5% proparacaine hydrochloride (Alcaine®, Alcon, Fort Worth, TX, USA) for anesthesia. Simultaneously, conjunctival swab was performed for all cases using a sterile cotton-tipped swab for thioglycolate broth. Scrapings were smeared on glass slides and Gram staining was performed. The cultured bacteria were identified using an automatic microbiological analyzer (VITEK 2 system; bioMérieux, Marcy l’Etoile, France). Antibiotic susceptibility testing was performed using the Kirby–Bauer disc diffusion method, and the minimum inhibitory concentration was determined using an automated microbiological analyzer.

All patients were treated topically with antibiotic eye drop (January 1998–October 2005: 0.5% levofloxacin, Cravit®, Santen, Osaka, Japan; November 2005–December 2017: 0.5% moxifloxacin, Vigamox®, Alcon, Fort Worth, TX, USA) with fortified topical antibiotics (2% tobramycin, 5% ceftazidime) and systemic antibiotics (second generation cephalosporins and aminoglycoside) before the microbiological results were obtained.

Treatment outcomes, including epithelial healing time, final visual acuity, complications (such as persistent epithelial defect, corneal perforation, or endophthalmitis), surgical intervention, and clinical outcome were reviewed. Presenting (initial) and final Snellen best corrected visual acuity (BCVA) scores were reviewed and converted to logMAR scale scores. The clinical outcomes were assessed at the end of three months or at the completion of treatment and classified into three groups (good, moderate, and poor), as defined by Green [11]. Good (moderate) outcome was defined as final VA of 6/12 or better (6/18–6/60), no complications or surgical intervention, and no decrease in VA during treatment. Poor outcome was defined as final VA worse than 6/60, decrease in VA during treatment, presence of complications, or requiring surgical intervention.

The data were statistically analyzed using the Statistical Package for the Social Sciences 20.0 (IBM, Armonk, NY, USA). The chi-squared and Fisher’s exact tests were used for categorical data. Independent *t*-tests were used for comparison of mean values. Statistical significance was defined as *p* < 0.05. The risk factors for poor clinical outcome were performed on the total cohort of PA and PP keratitis and analyzed using logistic regression. In the univariate analysis, an independent variable with *p* < 0.1 was included in the multivariate analysis, and a variable with a final *p* < 0.05 was considered a significant risk factor. This study was approved by the Institutional Review Board of the Yeungnam University Hospital, South Korea (file no. YUMC 2017–07-014) and complied with the principles outlined in the Declaration of Helsinki.

## Results

During the 20-year study period, 937 inpatient cases were diagnosed clinically as bacterial keratitis, of which 383/937 (40.9%) were culture positive. *Pseudomonas* species were found in 88 of 383 positive cultures (23.0%). The most common *Pseudomonas* species was PA (46/88, 52.3%), followed by PP (25/88, 28.4%), and other *Pseudomonas* species (17/88, 19.3%). After excluding polymicrobial infection, 33 cases of PA and 16 cases of PP were included in the study.

### Epidemiologic findings and predisposing factors

The epidemiology and predisposing factors are summarized in Table 1. The mean age was higher in the PP group than in the PA group with no significant difference (59.3 ± 18.9 vs 47.0 ± 24.4 years, *p* = 0.060). The most common age subgroup was the 20–30-year-old subgroup in the PA group and the ≥60-year-old group in the PP group. Symptom duration was significantly longer in the PP group than in the PA group (9.5 ± 7.8 vs 4.3 ± 3.9 days, *p* = 0.022). No significant differences were observed in sex, seasonal distribution, and duration of hospitalization between the PA and PP groups.

**Table 1:** Epidemiologic characteristics and predisposing factors of *Pseudomonas aeruginosa* and *P. putida* keratitis

Characteristics	*P. aeruginosa* (n = 33)	*P. putida* (n = 16)	*p*-value (x^2^-test)
Male sex	16 (48.5)	7 (43.8)	0.755
Age, mean ± SD, years	47.0 ± 24.4	59.3 ± 18.9	0.060^†^
Age subgroup			
0–19	2 (6.1)	1 (6.3)	1.000*
20–39	14 (42.4)	1 (6.3)	0.018*
40–59	4 (12.1)	6 (37.5)	0.060*
≥ 60	13 (39.4)	8 (50.0)	0.482
Symptom duration^‡^			
mean ± SD, days	4.3 ± 3.9	9.5 ± 7.8	0.022^†^
> 5 days	8 (24.2)	11 (68.8)	0.003
Duration of hospitalization^§^,			
mean ± SD, days	10.4 ± 6.4	8.6 ± 4.9	0.325^†^
Seasonal distribution			
Spring (Mar-May)	7 (21.2)	2 (12.5)	0.698*
Summer (Jun-Aug)	6 (18.2)	6 (37.5)	0.169*
Autumn (Sep-Nov)	15 (45.5)	3 (18.8)	0.069
Winter (Dec-Feb)	5 (15.2)	5 (31.3)	0.261*
Predisposing factors			
Corneal trauma	9 (27.3)	10 (62.5)	0.018
Contact-lens wear	12 (36.4)	0 (0.0)	0.005*
Previous OSD^#^	7 (21.2)	6 (37.5)	0.304*
Previous ocular surgery^¶^	5 (15.2)	3 (18.8)	1.000*
Previous topical antibiotics use	12 (36.4)	8 (50.0)	0.362
Previous topical steroid use	5 (15.2)	0 (0.0)	0.158*
Systemic disease**	10 (30.3)	7 (43.8)	0.354
No apparent cause	8 (24.2)	4 (25.0)	1.000*

*SD* standard deviation, *OSD* ocular surface disease

Values indicate numbers (proportion) unless otherwise noted

*The *p*-value was calculated using Fisher’s exact test

^†^The *p*-value was calculated using independent *t*-test

^‡^Interval from the onset of symptoms to the time of initial presentation

^§^The period from admission to discharge

^#^*P. aeruginosa* group included necrotizing scleritis (two cases), exposure keratopathy (two cases), neurotrophic keratopathy (one case), bullous keratopathy (one case), and previous corneal ulcer (one case); *P. putida* group included exposure keratopathy (two cases), pseudophakic bullous keratopathy (one case), leukoma adherence (one case), herpetic keratitis (one case), and recurrent erosion syndrome (one case)

^¶^*P. aeruginosa* group included cataract surgery (four cases) and pars plana vitrectomy due to proliferative diabetic retinopathy (one case); *P. putida* group included cataract surgery (two cases) and Ahmed valve implantation due to neovascular glaucoma (one case)

^**^P. aeruginosa group included hypertension only (four cases), diabetes mellitus (three cases), cardiovascular abnormality with hypertension (two cases), and cardiovascular abnormality only (one case). *P. putida* group included hypertension only (three cases), diabetes mellitus with hypertension (one case), hepatitis B with hypertension (one case), rheumatoid arthritis (one case), and atrial septal defect (one case)

The most common predisposing factor for PA was wearing CLs and that for PP was corneal trauma. The ratio of wearing CLs (36.4% vs 0%, *p* = 0.005) and corneal trauma (27.3% vs 62.5%, *p* = 0.018) showed a significant difference between the PA and PP groups. The ratio of previous OSD (37.5% vs 21.2%, *p* = 0.304) was higher in the PP group than in the PA group, with no significant difference. The details of previous OSD and previous ocular surgery are described in the Table 1. No significant differences were observed between the PA and PP groups in the ratio of previous ocular surgery, previous topical antibiotic use, previous topical steroid use, or systemic diseases.

### Clinical characteristics at initial presentation and treatment outcome (Table 2)

Both PA and PP groups had more central corneal lesions, with no significant difference between the two groups. No significant differences were observed in epithelial defect size between the PA and PP groups when the reference was ≥5 mm^2^. Hypopyon was significantly higher in the PA group than in the PP group (45.5% vs 6.3%, *p* = 0.006).

**Table 2:** Clinical characteristics and treatment outcomes of *Pseudomonas aeruginosa* and *P. putida* keratitis

Characteristics	*P. aeruginosa* (n = 33)	*P. putida* (n = 16)	*p*-value (x^2^-test)
Central lesion^‡^	24 (72.7)	12 (75.0)	1.000*
Epithelial defect size ≥5 mm^2^	21 (63.6)	9 (56.3)	0.619
Hypopyon	15 (45.5)	1 (6.3)	0.006
Epithelial healing time ≥ 10 days	20 (60.6)	9 (56.3)	0.771
Complications**	5 (15.2)	3 (18.8)	1.000*
Surgical treatment^++^	5 (15.2)	2 (12.5)	1.000*
Presenting BCVA^§^			
mean ± SD, logMAR	1.66 ± 0.95	1.53 ± 1.10	0.675^†^
< 0.1, Snellen	20 (62.5)	9 (56.3)	0.676
Final BCVA^§^			
mean ± SD, logMAR	0.98 ± 1.12	1.09 ± 1.22	0.760^†^
< 0.1, Snellen	9 (28.1)	6 (37.5)	0.509
Decreased BCVA^#§^	8 (25.0)	5 (31.3)	0.735*
Clinical outcome^§¶^			
Good	11 (34.4)	7 (43.8)	0.527
Moderate	11 (34.4)	3 (18.8)	0.328*
Poor	10 (31.2)	6 (37.5)	0.665

*BCVA* best corrected visual acuity, *logMAR* logarithm of the minimum angle of resolution, *SD* standard deviation

Values indicate numbers (proportion) unless otherwise noted

*The *p*-value was calculated using Fisher’s exact test

^†^The *p*-value was calculated using independent *t*-test

^‡^Corneal lesion is located within the 1/2 radius from the center of the cornea

**Complicated cases in *P. aeruginosa* group were persistent epithelial defect (two cases), impending corneal perforation (two cases), and progression of infection (one case); those in *P. putida* group was persistent epithelial defect (three cases)

^++^Surgical treatment cases in *P. aeruginosa* group were amniotic membrane transplantation (three cases), tarsorrhaphy (one case), and evisceration (one case); those in *P. putida* group was amniotic membrane transplantation (two cases)

^§^Total *n* = 48: One young child in the *P. aeruginosa* group who could not read letters was excluded

^#^Cases of final BCVA worsened from presentation

^¶^The clinical outcomes were assessed at the end of three months or at the completion of treatment and classified into three groups, as defined by Green [11].

More than half of the patients in both PA and PP groups had more than 10 days of epithelial healing time. There were no significant differences in the ratio of complications and surgical treatment between the PA and PP groups. All complicated cases in the PA group and two of three complicated cases in the PP group required surgical intervention. The details of complicated cases and surgical treatment cases are described in the Table 2.

In both PA and PP groups, final BCVAs improved significantly compared to presenting BCVAs (*p* = 0.006, *p* = 0.018, respectively). Mean presenting BCVAs, mean final BCVAs, the ratio of final BCVAs < 0.1 (snellen), and the ratio of decreased BCVAs at final visit were not significantly different between the PA and PP groups. The ratio of poor clinical outcome was 31.2% in the PA group and 37.5% in the PP group, with no statistically significant difference (*p* = 0.665).

### Antimicrobial susceptibility

The antibiotic susceptibility of both groups is shown in Table 3. Imipenem (100% vs 81.3%, *p* = 0.030) and piperacillin (96.6% vs 75%, *p* = 0.047) were significantly more effective in the PA group than in the PP group. Ticarcillin (85% vs 0%, *p* < 0.001) and ticarcillin/clavulanate (74.1% vs 0%, *p* < 0.001) were relatively effective against PA, but both were ineffective against PP. Colistin, tobramycin, meropenem, ceftazidime, ciprofloxacin, and levofloxacin showed good effectiveness in both PA and PP group.

**Table 3:** Antibiotics susceptibility of *Pseudomonas aeruginosa* and *P. putida* keratitis during the 1998–2017 period

Antibiotics	*P. aeruginosa* (n = 33)	*P. putida* (n = 16)	*p*-value (x^2^-test)
Beta-lactams			
Ticarcillin	17/20 (85.0)	0/7 (0.0)	< 0.001*
Tic/clav	20/27 (74.1)	0/16 (0.0)	< 0.001
Piperacillin	28/29 (96.6)	12/16 (75.0)	0.047*
Pip/tazo	18/19 (94.7)	12/16 (75.0)	0.156*
Carbenicillin	10/14 (71.4)	–	–
Amp/sul	0/17 (0.0)	0/12 (0.0)	–
Cefotaxime	0/28 (0.0)	1/16 (6.3)	0.364*
Ceftazidime	31/33 (93.9)	14/16 (87.5)	0.588*
Cefepime	30/31 (96.8)	15/16 (93.8)	1.000*
Aztreonam	16/29 (55.2)	1/16 (6.3)	0.001
Imipenem	33/33 (100.0)	13/16 (81.3)	0.030*
Meropenem	28/28 (100.0)	14/16 (87.5)	0.127*
Aminoglycosides			
Amikacin	30/33 (90.9)	16/16 (100.0)	0.541*
Gentamicin	30/33 (90.9)	15/16 (93.8)	1.000*
Tobramycin	14/15 (93.3)	7/7 (100.0)	1.000*
Netilmicin	2/2 (100.0)	3/4 (75.0)	1.000*
Isepamicin	2/2 (100.0)	3/4 (75.0)	1.000*
Fluoroquinolones			
Ciprofloxacin	28/29 (96.6)	14/16 (87.5)	0.285*
Levofloxacin	6/7 (85.7)	6/7 (85.7)	1.000*
Others			
Pipemidic acid	3/4 (75.0)	–	–
Tigecycline	2/16 (12.5)	1/9 (11.1)	1.000*
TMP/SMX	0/28 (0.0)	0/16 (0.0)	–
Colistin	18/18 (100.0)	15/15 (100.0)	–

Tic/clav = ticarcillin/clavulanate; Pip/tazo = piperacillin/tazobactam; Amp/sul = ampicillin/sulbactam;

TMP/SMX = trimethoprim/sulfamethoxazole

Values are presented as n1/n2 (%), where n1 is the number of isolates with susceptibility and n2 is the number of tested isolates

*The *p*-value was calculated using Fisher’s exact test

### Risk factor analysis of poor clinical outcomes

In the univariate logistic regression analysis, previous OSD, previous ocular surgery, nonuse of CLs, age of ≥50 years, epithelial healing time (≥10 days), and hypopyon were significant variables (all, *p* < 0.1). The multivariate logistic regression analysis of the significant variables revealed that previous OSD (odds ratio [OR] 10.79, 95% confidence interval [CI] 1.67–69.76, *p* = 0.012) and hypopyon (OR 9.02, 95% CI 1.33–61.25, *p* = 0.024) were statistically significant risk factors for poor clinical outcome (Table 4).

**Table 4:** Risk factors for poor clinical outcome* in *Pseudomonas* keratitis^†^ (univariate and multivariate logistic regression analysis^‡^)

Variables	Univariate analysis			Multivariate analysis		
	OR	95% CI	*p*-value	OR	95% CI	*p*-value
Hypopyon	3.57	0.98–12.97	0.053	9.02	1.33–61.25	0.024
Epithelial healing time ≥ 10 (days)	4.91	1.17–20.62	0.030	–	–	–
Age ≥ 50 (years)	5.57	1.33–23.44	0.019	4.85	0.74–31.68	0.099
Nonuse of CLs	7.86	0.91–67.57	0.060	–	–	–
Previous ocular surgery	9.00	1.56–51.95	0.014	6.48	0.72–58.65	0.097
Previous OSD	16.11	3.38–76.76	< 0.001	10.79	1.67–69.76	0.012

*CI* confidence interval, *CL* contact lens, *OSD* ocular surface disease, *OR* odds ratio

*The clinical outcomes were assessed at the end of three months or at the completion of treatment and classified into three groups, as defined by Green [11].

†Total cohort of *P. aeruginosa* and *P. putida* keratitis, total *n* = 48: One young child in the *P. aeruginosa* group who could not read letters was excluded

‡Multivariate logistic regression analysis was performed using the backward-conditional method for the factors with a *p*-value < 0.1 in univariate logistic regression analysis

## Discussion

The proportion of gram-negative bacteria among total bacterial keratitis has been increasing recently. Tam et al. reported that the proportion of gram-negative bacterial keratitis increased from 19% in 2000–2003 to 30% in 2012–2015 [12]. *Pseudomonas* species has continued to be the most common isolate of gram-negative bacterial keratitis; the proportion varies among countries, ranging from 10% of cases in North America [12–14], 24.4% in Taiwan [15], 32.5% in South Korea [16], and up to 52% in South India [17].

PP is a relatively rare pathogen of corneal infection and has been rarely reported. This study includes the largest number (16 eyes) of culture-proven PP keratitis*.* PP is known to be found in soil and water and is a rare source of human infection, causing nosocomial infections particularly in immunocompromised patients [18]. In this study, differences in epidemiologic and predisposing factors between the PA and PP group were identified. Compared to the PA group, the PP group had slightly higher ratio of compromised condition such as older age, previous OSD, and underlying systemic diseases, but with no statistically significant.

Many studies have reported wearing CLs as a common predisposing factor of bacterial keratitis [19, 20]. The close relationship between PA keratitis and CL wearing is well-known [21, 22]. In contrast, the studies of the association of PP keratitis with wearing CLs are rare reported, with only a case study of PP identified in orthokeratology-related cases [6]. In this study, CL wearing was the most common predisposing factor in the PA group, but there was no association between the PP group and CL wearing. The reason for the low detection of PP in CL wearing is not yet known, and further studies of the association between the PP and CL wearing are needed in the future.

The mean age of the PP group was higher than that of the PA group. In this study, we found that wearing CL was mainly restricted to younger ages, with all CL wearers aged < 50 years. When the CL wearers were excluded (PA 21 cases, PP 16 cases), the mean age showed no significant difference between the PA and PP groups (61.1 ± 18.9 vs 59.3 ± 18.9 years, *p* = 0.772).

The long interval of symptom duration is associated with delayed diagnosis and treatment, which has been reported to affect treatment outcomes. Fong et al. reported that delayed diagnosis and treatment of *Pseudomonas* keratitis may lead to subsequent failure of medical therapy with serious complications [23]. The mean symptom duration was longer in the PP group than in the PA group; even when the cases with CL wearers were excluded, the mean symptom duration was still longer in the PP group than in the PA group (9.5 ± 7.8 vs 5.5 ± 4.3 days, *p* = 0.077). In univariate analysis, symptom duration was identified as a significant factor for poor clinical outcome in the PA group (OR 1.26, 95% CI 1.01–1.57, *p* = 0.044) and was nonsignificant in the PP group (OR 1.11, 95% CI 0.95–1.30, *p* = 0.188). These results seem to be related to the characteristics of the PA keratitis showing rapid progression of corneal lesion from the early stage of keratitis and were interpreted that PP keratitis showed relatively slow progression compared to PA.

Among the clinical characteristics, hypopyon was significantly more common in the PA group than in the PP group, and no significant differences were observed in location of the corneal lesions or epithelial defect sizes between the two groups. When the cases with CL wearers were excluded, hypopyon was still more common in the PA group than in the PP group (57.1% vs 6.3%, *p* = 0.001); central lesion (76.2% vs 75%, *p* = 1.000) and epithelial defect size > 5 mm^2^ (71.4% vs 56.2%, *p* = 0.338) were nonsignificant. These results show that the clinical characteristics of PA keratitis are more severe than those of PP, even if the CL wearers group is excluded. These results can be explained by difference in virulence among *Pseudomonas* species [3]. The virulence of the PA is known to be due to proteases (alkaline protease, elastase A, and elastase B) and exotoxins, which actively digest corneal proteins and result in the degradation of the proteoglycan matrix [2, 24–26]. However, the associated corneal virulence factors including exotoxins and a secreted protease are absent in the PP [1, 2, 27].

Antibiotic susceptibility must be analyzed in relation to regional characteristics. In a study comparing the susceptibility of PA to ciprofloxacin, gentamicin, and cephalosporins by country, ciprofloxacin and gentamicin exhibited low resistance of under 10% in most countries for the period of 1990–2003. However, in India study resistance to ciprofloxacin reached 30% and resistance to gentamicin reached 46% (1992–2003 period). The ceftazidime resistance ranged from 0 to 14% by country [28]. In our study, ciprofloxacin, gentamicin, and ceftazidime demonstrated less than 10% of resistance in PA.

Decreased antibiotic susceptibility of PP to beta-lactams was observed in this study. Many other studies also reported beta-lactam resistance of PP in nosocomial infection [5, 29]. One of the resistance mechanisms reported was that clinical isolates of PP produce metallo-beta-lactamases, conferring resistance to beta-lactams such as carbapenems [30]. Among beta-lactams, the susceptibility of PP to cefepime (93.8%) and ceftazidime (87.5%), commonly used antibiotics for the treatment of bacterial keratitis, was relatively good in this study.

Because PA species is a more virulent than PP species, the clinical characteristics of the PA keratitis are expected more rapid progression and more severe clinical findings, and show worse outcome than PP keratitis. However, in this study, poor clinical outcome was observed at a similar rate between the PA and PP groups. Even when the cases with CL wearers were excluded, the ratio of poor clinical outcome was similar between the PA and PP group (45.0% vs 37.5%, *p* = 0.741). This result may be related to the higher ratio of previous OSD and elderly age in the PP group than that of PA group, which are risk factors for poor clinical outcome. In addition, this result show that the PP species can also be a causative pathogen of severe keratitis. Therefore, clinicians should pay attention to the clinical significance of PP keratitis in patients with these factors.

The significant risk factors for poor clinical outcome were previous OSD and hypopyon. Several studies have reported previous OSD as prognostic factors of bacterial keratitis [11, 31]. It is thought that the treatment outcomes of patients with previous OSD could be affected by the preexisting disease itself. Hypopyon in bacterial keratitis comprises sterile leukocytic exudate that is associated with severe inflammation. A relationship has been reported between hypopyon and treatment outcome; Lavinsky et al. found that the degree of hypopyon was significantly related to the length of hospital stay and worse visual outcome in patients admitted for bacterial keratitis [32].

Our study had some limitations. First, this study was retrospective and had a limited number of included cases. Second, the included patients were confined to inpatients in a tertiary referral center and might have had more severe symptoms compared with outpatients or those at a primary medical clinic. Therefore, the results of this study are limited to those of patients at tertiary hospitals. Third, this study was performed at a single center; therefore, our results may not represent nationwide data of South Korea. A future study using multicenter data is required. Despite these limitations, this study provides epidemiological data, predisposing factors, clinical characteristics, and antibiotic susceptibility of PP which is a rare pathogen causing bacterial keratitis. Another strength of this study is that it enabled the assessment of clinical characteristics of monomicrobial infection by excluding factors associated with other pathogens in polymicrobial infection.

## Conclusions

In conclusion, PA keratitis was more closely associated with young age and CL wear, and PP keratitis was more closely associated with elderly age and corneal trauma. The efficacy of beta-lactams in the PP group was lower than that in the PA group. The PA group had shorter symptom duration and more severe clinical characteristics, such as hypopyon, than the PP group, but the clinical outcomes were not significantly different between the two groups. The risk factors for poor clinical outcomes of *Pseudomonas* keratitis were previous OSD and hypopyon. This study provides information on the differences in clinical characteristics and antibiotic susceptibility between patients with PA and PP keratitis in South Korea.

## Abbreviations

BCVAs: Best corrected VAs
CI: Confidence interval
CL: Contact-lens
OR: Odds ratio
OSD: Ocular surface disease
PA: *Pseudomonas aeruginosa*
PP: *Pseudomonas putida*
VA: Visual acuity
